# Understanding reproductive health challenges during a flood: insights from Belkuchi Upazila, Bangladesh

**DOI:** 10.12688/gatesopenres.12920.2

**Published:** 2019-06-28

**Authors:** Nibedita S. Ray-Bennett, Denise M. J. Corsel, Nimisha Goswami, Aditi Ghosh

**Affiliations:** 1 School of Business, University of Leicester, Leicester, LE1 7RH, UK; 2 International Planned Parenthood Federation (IPPF) - South Asia Region Office, New Delhi, 110049, India; 3 International Planned Parenthood Federation (IPPF), Bangkok, 10900, Thailand

**Keywords:** Belkuchi, Bangladesh, menstrual regulation, post-abortion care, health facilities, flood, women, RHCC.

## Abstract

**Background: **Bangladesh is exposed to natural hazards such as floods, cyclones and droughts. As such, its health systems and health infrastructure are exposed to recurrent disasters. Research studying the impacts of natural disasters on reproductive health in particular is lacking. This research contributes to this knowledge gap by studying the challenges related to menstrual regulation and post-abortion care at both the facility and community levels, and the care-seeking patterns of pregnant women during the 2016 flood in Belkuchi, Bangladesh.

**Methods: **Six government-run primary health care facilities were assessed using a structured assessment tool prior to the flood of 2016. In total, 370 structured interviews were conducted with women in three unions of Belkuchi (Belkuchi Sadar, Daulatpur and Bhangabari) 4 months after the 2016 flood.

**Results: **The main challenges at the facility level are a lack of services and a shortage of medicines, equipment and trained health workers. The main challenges at the community level are displacement, high rates of self-diagnosed spontaneous abortion and a lack of treatment for post-abortion complications. A majority of the interviewed women (48%) sought menstrual regulation from the residence of a nurse or family welfare visitor. In total, 73.2% of the women who experienced post-abortion complications sought medical care.

**Conclusion: **To overcome the challenges at the facility level, it is important to construct flood-resistant health infrastructure and train health workers in menstrual regulation and post-abortion care, so that these services can be made available during a flood. At the community level, more research is required to understand the reasons for spontaneous abortions so that these, and the subsequent chronic conditions/complications women experience, may be avoided. Context specific interventions that can overcome local challenges (both at the community and facility levels) are required to promote disaster resilience at primary health care facilities.

## Introduction

Bangladesh is exposed to natural hazards due to its geographic location. The World Bank report on *Natural Disaster Hotspots* ( Dilley *et al*., 2005) highlighted that Bangladesh is in the top 60 countries of the world that are highly prone to two or more hazards (flood, cyclone, storm and drought). It is estimated that approximately 97.1% of Bangladesh’s total area is at risk of two or more hazards, putting 97.7% of the population at risk. This estimate puts Bangladesh as the number one country in the world relative to mortality risk from two or more hazards. Additionally, due to climate change, Bangladesh is predicted to experience natural disasters more frequently, and inundation of 10% of its land mass due to rising sea levels ( WHO, 2012). This is likely to cause a loss of agricultural land, an increase in homelessness and displacement and tremendous pressure on health systems and health infrastructure ( IPCC, 2018; WHO, 2012). An increase in the frequency of disasters due to global warming and climate change, and their anticipated deleterious effects on poor nations such as Bangladesh, is likely to create a complex and challenging environment for health systems, particularly those in rural areas, in the coming years.

Despite severe physical and environmental challenges, Bangladesh is one of the few developing countries to have met its target for Millennium Development Goal 5 by reducing the maternal mortality ratio from 322 deaths per 100, 000 live births in 1998–2001 to 176 deaths per 100,000 live births in 2013 ( El Arifeen *et al*., 2014; Gideon *et al*., 2015; WHO, 2015b). Although this is a remarkable achievement, reducing maternal mortality is still a challenge, as is improving maternal health from unsafe abortions and post-abortion complications in general, and especially so during disasters ( Ahmed *et al*., 2011; Huda *et al*., 2013). The UN’s ‘Global Strategy for Women’s, Children’s and Adolescents Health’ and the Sustainable Development Goals have both set an ambitious target for all the nation-states of ending preventable maternal deaths by 2030. This means reducing maternal deaths to fewer than 70 per 100,000 live births ( Koblinsky *et al*., 2016; UNFPA, 2015).

In order to reduce maternal mortality and morbidity from miscarriages, unsafe abortions and post-abortion complications for Sustainable Development Goal 3, it is important to understand the challenges related to menstrual regulation and post-abortion care at facility and community levels, and the care-seeking patterns of pregnant women during a flood. In light of this, the research questions for this study are: what are the challenges at the facility level with regard to menstrual regulation and post-abortion care during a flood, and what are women’s care-seeking patterns? The research questions are addressed through a case study from Belkuchi Upazila, a flood prone sub-district of Sirajganj District in Bangladesh. Context specific challenges are also highlighted to inform appropriate measures to promote women’s reproductive health and wellbeing, and to build a ‘disaster resilient health system’ to support sustainable development ( UN, 2015; UNFPA, 1995; UNFPA, 2016).

### Menstrual regulation and post-abortion care

Menstrual regulation is defined as ‘evacuation of the uterus performed by a trained provider’ ( Huda *et al*., 2013: 10) within 12 weeks of a missed period using manual vacuum aspiration or a combination of Mifepristone and Misoprostol medication ( Guttmacher Institute, 2017; Yasmin *et al*., 2015). Post-abortion care is a set of interventions used to reduce injuries and deaths from incomplete and unsafe abortions, as well as to address any complications that may arise ( Ipas, 2018). According to Ipas (2018), post-abortion care includes five essential elements: i) treatment, ii) counselling, iii) contraceptive and family-planning services, iv) reproductive and other services, and v) community and service-provider partnerships.

Menstrual regulation is a nation-wide family planning programme. It was first introduced by the Government of Bangladesh in 1974 within government clinics in an attempt to reduce the rate of maternal mortality and morbidity due to complications from septic abortion ( Kay & Kabir, 1988; WHO, 2015a). By 1979 the government approved menstrual regulation as an ‘interim method of establishing non-pregnancy’ ( Yasmin *et al*., 2015) and it was integrated into the national family planning programme. Although abortion is illegal, menstrual regulation is legal in Bangladesh up to 12 weeks ( Guttmacher Institute, 2017; UN, 2001) and these services are provided through a partnership between the Directorate General of Family Planning and a key group of non-governmental organisations. Post-abortion care services are provided under the ambit of the Directorate General of Health Services and the Directorate General of Family Planning facilities ( Biswas *et al*., 2013; Biswas *et al*., 2017). Although there is an administrative division between the menstrual regulation and post-abortion care services, they are both provided at the Upazila Health Complex through its two wings: the family planning wing for menstrual regulation and the health wing for post-abortion care ( Biswas *et al*., 2013; Huda *et al*., 2015).

There is a plethora of studies that have investigated and evaluated the challenges and opportunities related to the availability, accessibility ( Biswas, *et al*., 2013; Huda *et al*., 2013; Nasreen *et al*., 2010; Yasmin *et al*., 2015) and quality of this nation-wide family planning programme ( Huda *et al*., 2015; Kay & Kabir, 1988; Vlassoff *et al*., 2012; WHO, 2015b). There are also several reliable national data sets that provide a clearer picture of menstrual regulation and unsafe abortion based on health facility surveys ( Guttmacher Institute, 2017; Vlassoff *et al*., 2012). Researchers have also studied the role of social networks ( Gyen & Raeside, 2007; Gyen & Raeside, 2010), decision making at household levels ( Story & Burgard, 2012) and voucher programmes ( Nguyen *et al*., 2012) to promote the use of these services. Research studying the challenges related to menstrual regulation and post-abortion care during disasters, including floods in Bangladesh, is lacking. As such, this study is both novel and timely.

### Primary health care facilities

The focus of this research project is on the primary health care system. At the International Conference on Primary Health Care in 1978, the Declaration of Alma-Ata conceived primary health care as the “essential health care based on practical, scientifically sound and socially acceptable methods and technology made universally accessible to individuals and families in the community through their full participation and at a cost that the community and country can afford to maintain” ( WHO, 1978: 1–2). More recently, the WHO (2018) highlighted primary health care as being “about caring for people, rather than simply treating specific diseases or conditions”. The core principles of primary health care include: i) universal access; ii) community participation in defining and implementing health agendas; iii) intersectoral approaches to health; and iv) commitment to health equity ( WHO, 2013). Many scholars and institutions have developed and added to these principles. For instance, VON Canada (2018) have adopted the first three core principles and added ‘appropriate use of technology’ and ‘health promotion’. Fundamentally all the principles indicate that primary health care involves making essential health care services available to everyone, including the poor and vulnerable, in the community ( Kendall, 2008; VON Canada, 2018; WHO 2018). Primary health care is then the first point of contact that people have with the health system. This care is integral for a community’s wellbeing. It is also important that everyone can access this health system for the services they require ( Bryar, 2000; Kendall, 2008; VON Canada, 2018; WHO, 2018). This is because the Alma-Ata Declaration affirms that access to health care is a matter of human rights and it is through the provision of primary health care that this can be achieved ( Kendall, 2008; WHO, 1978).

**Figure 1.**
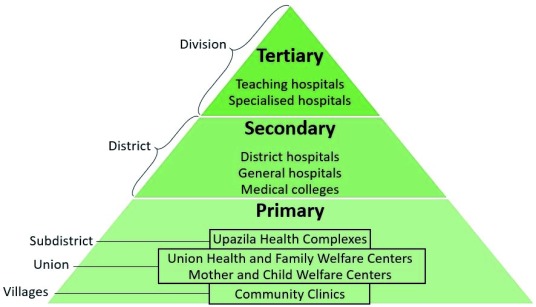
Health care system in Bangladesh.

Bangladesh has signed to the Alma-Ata Declaration and included the primary health care approach in the nation’s First Five Year Plan (1972–1978). It implemented this Declaration by establishing Upazila Health Complexes ( WHO, 2008; WHO, 2015b). Bangladesh has a decentralised health care system (see Figure 1) in order to make services available to everyone ( Kouam *et al*., 2014; WHO, 2015b). The primary health care system in Bangladesh consists of: Upazila Health Complexes, Union Health and Family Welfare Centers and Community Clinics ( Kouam *et al*., 2014; WHO, 2015b). This decentralised system is seen as efficient because the facilities are the first point of contact for rural communities and they cover the entire spectrum of health care services. These facilities often fulfil similar roles to District Hospitals ( Abbas & Routray, 2013). Studies in Bangladesh and elsewhere have also confirmed the vital role that primary health care facilities play during floods ( Abbas & Routray, 2013), as well as in reducing mortality and morbidity in order to meet the nation’s target for Millennium Development Goal 5 through the provision of family planning services, contraception and basic essential health care services ( El Arifeen *et al.,* 2014; Gideon *et al*., 2015; WHO, 2015b).

Despite the above achievements, there are concerns about the outreach of primary health care in rural areas in Bangladesh ( Islam & Biswas, 2014). Proponents argue that primary health care is not adequately integrated into the national health system because government-run hospitals are ‘often inaccessible, crowded, understaffed and lacking medicines’ ( WHO, 2008). They are under-funded and facilities are poorly stocked with medical instruments, devices and supplies ( WHO, 2015b). In the context of our study this also extends to a shortage of skilled staff.

As mentioned above Bangladesh is vulnerable to natural hazards such as floods, droughts and cyclones ( Azad *et al*., 2013; WHO, 2012). As such, primary health care facilities are also exposed to natural hazards and disasters ( WHO, 2011). The physical vulnerability of the health care facilities is of great concern as it can hinder or even cease the delivery of essential health care services ( WHO, 2011). For instance, flooding can cause structural failure (such as damage to infrastructure, medical equipment, power supplies, communication means, transportation methods and water supplies), which can inhibit the health facility’s operation ( Axelrod *et al*., 1994; Phalkey *et al*., 2012; Van Minh *et al*., 2014). Therefore, it is important that the primary health care system is prepared to address the ‘Sendai Framework for Disaster Risk Reduction’s’ Global Target D: “Substantially reduce disaster damage to critical infrastructure and *disruption of basic services* [authors emphasis], among them health and educational facilities, including through developing their resilience by 2030” ( UN, 2015: 12).

A resilient primary health care system should aim to continue providing services (including reproductive health services) in disaster situations, with minimal disruption. However, developing a resilient health system is a challenge for countries with ‘overstretched-staff and weak governance’ ( Koblinsky *et al*., 2016). ‘Increasing and optimising health workforce’ and ‘improving facility capability’ for resilience building ( Koblinsky *et al*., 2016) demands multiple interventions from multiple actors and a long-term financial commitment from the Government of Bangladesh and international donors. This study, the first of its kind, aims to highlight some of the challenges of primary health care facilities and of pregnant women, so that they may inform policy makers and international practitioners and enhance disaster resilience.

## Methods

### Sample selection

The research location, Belkuchi Upazila, was chosen after consulting with the former Director of the Comprehensive Disaster Management Program and the Disaster Management Response Specialist from the Department of Disaster Management of Bangladesh ^1^. Belkuchi is one of the nine upazilas (sub-districts) of Sirajganj district located in the northern part of Bangladesh. It covers an area of 164.31 sq. km. ( Upazila Disaster Management Committee, 2014). According to the 2011 census, there were 157 villages, 74,450 households and a total population of 352,835 (173,097 of which are female) in Belkuchi. Belkuchi has six unions, which are Belkuchi Sadar, Bhangabari, Daulatpur, Bordhul, Dhukuriabera and Rajapur.

Belkuchi Upazila is among the most vulnerable and disaster-prone area in Sirajganj district ( Upazila Disaster Management Committee, 2014). It sits on the floodplain of two rivers, Jamuna and Hursagar. Due to its unique geographic location, Belkuchi gets flooded annually. A review of the location’s disaster data suggested that Belkuchi has been flooded every year, but that the severity varies; for instance, the floods of 1988, 1998, 2004 and 2007 were remarkably worse ( Upazila Disaster Management Committee, 2014). It was therefore an ideal research location. From July to August 2016, Belkuchi experienced heavy rainfall and riverine flooding ( Dhaka Tribune, 2016a). The floods created immense challenges, especially for vulnerable pregnant women ( Dhaka Tribune, 2016b).

In Belkuchi, the Upazila Health Complex is the only designated public facility for obstetric and gynaecological care. There are also five Union Health and Family Welfare Centers designed to cater for menstrual regulation and post-abortion care services. All these government-run facilities were selected for assessment. Approval was sought and received from the Directorate General of Family Planning (Memo Number: DGFP/MCH-S/icddr,b-2/09/(Part-1)/753) on 24 July 2016 and from the Directorate General of Health Services (Memo Number: DGHS/MNCAH/MNH/2016/271) on 16 August 2016, which are under the ambit of the Ministry of Health and Family Welfare of Bangladesh. The approval from the two wings of the Ministry of Health and Family Welfare secured cooperation from the Belkuchi Upazila Health Complex and the Union Health and Family Welfare Centers to undertake the facility assessments.

### Facility assessments

The assessments were conducted by staff from the International Centre for Diarrhoeal Disease Research, Bangladesh (icddr,b) on the 23 ^rd^ and 24 ^th^ of July 2016, the day after the monsoon season started ( Reliefweb, 2016), and so any changes that have occurred in these facilities since the assessments are not reflected. The quality of the six designated menstrual regulation and post-abortion care public facilities were assessed using a structured assessment tool, which included reviewing: i) human resources; ii) menstrual regulation and post-abortion care management within the facility; iii) menstrual regulation and post-abortion care related service delivery performance; iv) logistics and medical equipment; v) essential drugs/solutions for post-abortion care; and vi) needs assessment for menstrual regulation and post-abortion care training. The resources that the structured assessment tool assessed are available on OSF ( Ray-Bennett *et al*., 2019). Information on these facilities was also gained by consulting with 10 health workers. These health workers included one resident medical officer, five sub-assistant community medical officers and four family welfare visitors ^2^.

### Interviews

Four months after the flood (January–February 2017) structured interviews were conducted in three unions: Belkuchi Sadar, Bhangabari and Daulatpur. These unions are highly prone to flooding due to its low land and the absence of flood protection dams ( Upazila Disaster Management Committee, 2014). To conduct theses interviews, approval was sought from the University of Leicester’s Ethics Sub-Committee for the Media and Communication and School of Management (Ethics Reference: 8984-nsrb1-schoolofmanagement), and from icddr,b’s Research Review Committee and Ethical Review Committee in Dhaka (Ethics Protocol Number: PR-16079). All participants were informed of the research aims and objectives. Informed consent was sought from all the participants who partook in the interviews. This was done in the form of verbal consent to ensure that all participants fully understood, despite their literacy. No names or identifiable details were used, and the participants were coded for anonymity. All participants were told that they could stop participation at any given point, without providing a reason.

A total of 8,862 women were screened in Belkuchi Sadar, 9,905 women in Bhangabari and 9,809 in Daulatpur to try to receive an approximate equal sample size in each union. The goal was to have a 10% sample size, but we were able to screen more women. Subsequently, with a population size of 105,725, a sample size of 28,576 and a confidence level of 95%, the margin of error was 0%. In total 28,576 women (approximately 27% of the female population in the three unions) were screened, of which 372 women met the interview selection criteria. The structured interviews were deliberately conducted 4 months after the 2016 flood (from January- February 2019) in order to give the community some time to recover from the flood and any medical procedures they may have had. Furthermore, it was important to wait for the streets and houses to dry for the convenience of the fieldworkers to conduct the structured interviews. Muddy and waterlogged streets and alleys in between houses and neighbourhoods become impassable immediately after a flood.

The participants for the structured interviews were selected through a multi-stage criterion-based sampling strategy. First, this included selecting three out of the six unions in Belkuchi based on physical convenience. Belkuchi Sadar, Bhangabari and Daulatpur unions were purposively selected because of their close proximity to each other. Second, the three sampled unions were divided into 68 clusters having a more or less an equal population size, of which 41 were randomly selected for structured interviews. Third, six female field research assistants made door-to-door visits to create a list of all of the women residing in the 41 clusters in order to screen participants. Since the record keeping of menstrual regulation and post-abortion care services was poor at the facilities, it was necessary to select participants through door-to-door home visits. The screening criteria were: i) married woman aged 15–49 years; ii) was staying in this area during the flood in 2016; and iii) received menstrual regulation and post-abortion care services during the last flood. In total, as mentioned above, 28,576 women were visited, of which 372 met the screening criteria.

Of the 372 women who met the screening criteria, two did not consent; hence, 370 were interviewed using a structured questionnaire. The interviews were conducted by six experienced and trained female field research assistants and it was ensured that they did not have any prior connections to the women that they would interview. These field research assistants were specifically selected to reduce any influence that they could have on the women being interviewed. The interviews lasted approximately 30 minutes to 2 hours, depending on the women’s response times and willingness to elaborate. The questionnaire of the structured interview was translated into Bengali and included the following sections: i) Socio-economic characteristics of our population; ii) Hazards, risks and vulnerability; iii) Knowledge of menstrual regulation and post-abortion care; iv) Care-seeking patterns for menstrual regulation and post-abortion care; and vi) Self-reported morbidities occurring during the flood. Although the questionnaire consisted of closed questions, there were a few open-ended questions in order to gain more detailed information. A sample of the survey questions used is available on OSF ( Ray-Bennett *et al*., 2019).

### Data processing and analysis

The data was imported into IBM SPSS Statistics version 24 for analysis. Since the analysis of the data was targeted towards discovering the menstrual regulation and post-abortion care related challenges during the flood, selective analysis was conducted by exploring each question individually and in-depth. Univariate and bivariate analysis was mainly used because most of the questions were independent and only some of the variables were comparable against each other. To summarise the variables, descriptive statistics, including frequencies, percentages, measures of central tendency and measures of variability were used. For the nominal variables, frequency tables were developed and the mode and mean were calculated. For the measurement variables, the same was done but also the range and median were calculated. Each of the calculations were cross-checked by a second member of the team to enhance the credibility and accuracy of data analysis. This level of analysis met the objectives of the research by providing sufficient insights into the menstrual regulation and post-abortion care situation in Belkuchi.

### Limitation of the data analysis

Facility assessments and interviews were selected as the most appropriate methods in order to explore the challenges related to menstrual regulation and post-abortion care at both the facility and community levels. The data collected from these methods were analysed descriptively. It is acknowledged that from this type of analysis, the data and findings cannot be generalised to the full area of Bangladesh. A comparative analysis to another area would have allowed for this but due to time and budget constrains this was not achievable. It is suggested, however, that the data is representative of the situation in Belkuchi, as well as other flood-prone upazilas with similar demographics. Additionally, the data can be used to raise awareness and identify the resources that will be required to improve the quality and uptake of public facilities.

## Results

### Challenges at facility level

*Lack of services*: From the interviews with the family welfare visitors during the facility assessments, it was found that the menstrual regulation and post-abortion care services are generally not provided at the five Union Health and Family Welfare Centers for three reasons:

i) Absence of trained family welfare visitors. Except for one of the Union Health and Family Welfare Centers, the other five Centers had newly appointed family welfare visitors. They were untrained and as such were unable to provide menstrual regulation and post-abortion care services.
ii) Religious beliefs of a few of the health workers deterred them from performing menstrual regulation procedures. One family welfare visitor stated: *“We perform MR [menstrual regulation], our tickets to Jahannam [hell] is ready because we are killing jans [foetuses].”* This was a cause for concern and subsequently, upon further exploration it was discovered that the health workers who were unwilling to perform menstrual regulation would advise their patients to go to the Upazila Health Complex instead.
iii) Motivation to increase the uptake of contraception acted as a barrier to menstrual regulation. Family welfare visitors often show reluctance to provide menstrual regulation services to increase the uptake of contraception. According to a family welfare visitor: “ *Many women prefer MR rather than taking pills regularly. Their uterus become vulnerable due to having repeated MR. This also increases the chance of maternal morbidity and mortality. I counsel women to use contraceptive methods and discourage to perform MR.*”

The facility assessments revealed that the catchment area for Belkuchi Upazila Health Complex is the whole Belkuchi Upazila and covers an area of 164.31 km ^2^. The total population of Belkuchi was 352,835 ( Upazila Disaster Management Committee, 2014). The catchment areas for the Union Health and Family Welfare Centers are smaller. Bordhul and Dhukuriabera are farthest from the Upazila Health Complex and are ‘hard to reach’ locations. Bordhul is a *chor* (island formed from silt) in the basin of the Jamuna River and thus, during floods, this area becomes almost inaccessible. Of all the primary health facilities available, women of all unions seek menstrual regulation and post-abortion care services in the Upazila Health Complex both in the wet and dry seasons.

**Figure 2.**
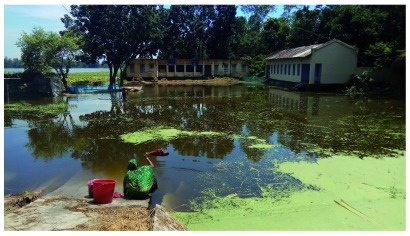
The start of the monsoon season at Daulatpur Union Health and Family Welfare Center in July 2016.

*Shortage of medicines and equipment*: The facility assessments revealed that the capacity of the Belkuchi Upazila Health Complex has been expanded to accommodate 50 indoor beds (from 31 beds) due to the addition of a new building (inaugurated in July 2017). Within Belkuchi, the only emergency obstetric care trained personnel were four nurses at the Upazila Health Complex. The Upazila Health Complex provided a number of family planning measures such as oral contraceptive pills, condoms, intra uterine devices, injections, implants and the emergency contraceptive pill. The supply of all Misoprostol medicines was withheld for the previous three months for unknown reasons. Of the 31 important pieces of equipment related to menstrual regulation and post-abortion care services (see OSF for the list of equipment/resources assessed by the structured assessment tool ( Ray-Bennett *et al*., 2019)), the Upazila Health Complex was rated 84% equipped and the Union Health and Family Welfare Centers in Bhangabari were rated 70.9%, Daulatpur 67.7%, Rajapur 67.7%, Dhukuriabera 45.2% and Bordhul 19.4%. The operating theatre lights were not functioning both at the Upazila Health Complex and at the Rajapur Union Health and Family Welfare Center during the time of the assessments. General counselling was provided at the Upazila Health Complex and the five Union Health and Family Welfare Centers. Post menstrual regulation/post-abortion contraceptive counselling was available in all the facilities, except in Bordhul. All the facilities were physically vulnerable to floods (for instance, see Figure 2).

### Challenges at the community level

Of the 370 participants, 34.1% were from Belkuchi Sadar, 34.1% were from Daulatpur and 31.9% were from Bhangabari. 93.5% of the participants were Muslim and 6.5% were Hindus. No other religious backgrounds were reported in the surveys. Overall, 59.7% of the participants had menstrual regulation services, while 40.3% received post-abortion care services during the flood of 2016.

*Displacement:* During the flood, 18.6% of the participants said that that they were displaced. The displaced participants went to a relative’s house (65.22%), neighbour’s house (7%), set up a camp on the road-side (13%) or on the embankment (10%). None of the participants went to a flood shelter despite 15.1% mentioning that there was a flood shelter near to their house. According to the participants the flood shelters lacked water supply and health care facilities.

*Spontaneous abortion*: In total, 53% of the participants reported that the outcome of their most recent failed pregnancy was self-reported ‘spontaneous abortion’, while 47% answered ‘menstrual regulation/induced abortion’.

*Complications:* After receiving the menstrual regulation, 23% of participants experienced complications, which were: severe or increased pain in lower abdomen; excessive bleeding more than two weeks; bleeding more than normal menstrual bleeding; weakness; medicine was not effective; vertigo; product did not come out; fever continued for more than one day; irregular menstrual cycle; headache; and nausea/vomiting.

*Access:* Without public transport and boat services, access to the primary health care facilities was a challenge during the flood of 2016.

**Figure 3.**
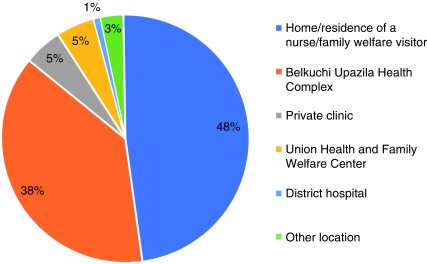
Location of receiving menstrual regulation during the 2016 floods.

***Women’s care-seeking patterns.*** Only 66 participants out of the 370 answered the question: ‘Where did you receive the healthcare services for your most recent menstrual regulation?’ The majority of the participants (48.48%) received the menstrual regulation from the home/residence of a Nurse or Female Welfare Visitor, followed by 37.88% from Belkuchi Upazila Health Complex, 4.55% from a Private Clinic, 4.55% from a Union Health and Family Welfare Center, 3.03% from ‘Other’ location and 1.52% from the District Hospital (see Figure 3).

For the menstrual regulation related complications mentioned above, 73.2% of the participants received healthcare services, while 3.2% did not; a further 23.5% did not respond to this question. Reasons for not receiving healthcare were:

*“The cost of the services was too high”*
*“I was afraid to receive the service*”.

Only 44.6% of the participants were told when to return to the health facility for a follow up. 51.9% were not told, while 1.4% said that they cannot remember whether they were told or not. After the floods, only 11.2% of participants went back to the same facility to receive family planning methods. The most popular method was IUD/Copper-T, followed by ‘Oral contraceptive pill’ and the ‘Birth control injection’.

## Discussion

### Overcoming challenges at the facility level

The main challenges that were found at the facility level during both dry and wet seasons were: non-availability of menstrual regulation and post-abortion care services at the five Union Health and Family Welfare Centers; a shortage of trained health workers; religious barriers; and shortage of medicines and equipment. As such the utilization rates for these facilities were relatively low (42.43%).

Menstrual regulation and post-abortion care services were unavailable at all the five Union Health and Family Welfare Centers. As a result, women in Belkuchi had to travel and seek these services from the Upazila Health Complex in both wet and dry seasons. The facility assessments revealed that the technical quality of menstrual regulation and post-abortion care services in the Upazila Health Complex is adequate but not all of the physically measurable attributes meet acceptable standards. There is significant room for improvement.

It was observed that the Upazila Health Complex infrastructure is expanding and that their facilities are catering to the public’s demand. For instance, a new building with the capacity for 50 indoor beds has been inaugurated in July 2017. The facility assessments revealed that the Upazila Health Complex was well equipped (84%) in terms of human resources, medical devices, equipment, medicines and sterilisation facilities to carry out menstrual regulation and post-abortion care services and had a range of family planning measures. Compared to Bhangabari (70.9%), Daulatpur (67.7%) and Rajapur (67.7%) Union Health and Family Welfare Centers, Dhukuriabera (45.2%) and Bordhul (19.4%) Union Health and Family Welfare Centers were the least equipped. The latter two health facilities are the farthest from the Upazila Health Complex and are in *chor* areas; they get severely affected by floods. These facilities deserve special attention from the Ministry of Health and Family Welfare through funding, a steady supply of medicines and skilled staff ( Huda *et al*., 2013).

The Upazila Health Complex had counselling services, including post menstrual regulation and post-abortion contraceptive counselling. This is very important to maintain the overall quality of the menstrual regulation and post-abortion care services and to reduce future complications or reoccurrence. However, here are a few areas of concern at the Upazila Health Complex that reduce the quality of menstrual regulation and post-abortion care services and that require attention: i) hygiene is sometimes not maintained properly in the area/room where menstrual regulation and post-abortion care services are provided ^3^. This can cause infections, spread of diseases/bacteria, and increase in morbidity; ii) untrained health workers continue to provide menstrual regulation and post-abortion care services and this malpractice has a huge consequence in decreasing patient safety, satisfaction and good practices; and iii) shortage of medical supplies and devices. Each of these three challenges deserve urgent attention from the Ministry of Health and Family Welfare and the Upazila Health Complex management team. Close monitoring, incentives to maintain hygiene, training health workers and providing a steady supply of medicines and equipment, can help to overcome these challenges locally.

Religious beliefs that hinder menstrual regulation procedures are an equally serious challenge to reproductive health services. Studies elsewhere in Bangladesh also noted similar practices ( Huda *et al*., 2013; Vlassoff *et al*., 2012). Many family welfare visitors refuse to administer menstrual regulation and post-abortion care because it conflicts with their religious anti-abortion beliefs. Although menstrual regulation is legal the social stigma attached to the procedures is widespread ( Huda *et al*., 2015). Older cohorts of family welfare visitors who were trained to provide menstrual regulation are retiring, and there have not been enough new workers trained to replace them ( Huda *et al*., 2013; Vlassoff *et al*., 2012). Subsequently, as seen in this research, new family welfare assistants were untrained in menstrual regulation and the old family welfare visitors who are trained were not conducting the procedure for religious reasons. As a result, all the menstrual regulations were conducted only at the Belkuchi Upazila Health Complex by the untrained nurses. In order to counteract anti-menstrual regulation sentiments and the shortage of trained staff, it is crucial that the Ministry of Health and Welfare invest in continuous professional development courses and Value Clarification Attitude Transformation (VCAT) training for the different groups of health workers in order to challenge deep seated religious beliefs, which currently hinder menstrual regulation procedures in the Union Health and Family Welfare Centers. It is also important that the Upazila Health Complex management team creates a culture of reporting, one in which the existing family welfare visitors, family welfare assistants, nurses and other health workers can come forward to report their reservations for the menstrual regulation procedures so that an effective referral system can be put in place. This referral system should help pregnant women find a suitable health worker or a different health facility with no extra cost.

### Overcoming challenges at the community level

The main challenges women faced during the 2016 floods were: displacement; spontaneous abortion; and medical complications after receiving menstrual regulation and post-abortion care.

According to the Belkuchi Upazila Disaster Management Plan ( Upazila Disaster Management Committee, 2014), there are 3 flood shelters in Belkuchi, 96 public buildings as potential flood shelters (e.g. schools) and 8 government/NGO shelters. These shelters were not enough to provide refuge to the 100,000 people estimated to be marooned in the floods of 2016 ( Dhaka Tribune, 2016a). None of the participants went to the flood shelters. Reasons for not moving to flood shelters are not only the lack of shelters, but also that the shelters are ill-equipped to provide for the reproductive health needs of women. They lack toilet facilities and safe drinking water, as mentioned by our participants. They also lack facilities to cook, lactate, sleep and shower. Furthermore, these public-private spaces are often inconsistent with cultural practices of purdah (which creates a strict separation between men and women) ( Rashid & Michand 2000; Ray-Bennett, 2009; Story & Burgard, 2012). As such, displaced participants took refuge in their neighbours’ or relatives’ houses, or set up camp on the road-side or the embankment. To reduce the distress and suffering of pregnant women during a flood evacuation it is suggested that governmental and non-governmental organisations must include reproductive health as an integral part of relief and response activities. Sexual and reproductive health should also form an integral part of the management of flood shelters in order to increase their utility.

Of our participants, 53% answered that the outcome of their most recent failed pregnancy was ‘spontaneous abortion’, while 47% answered ‘menstrual regulation/induced abortion’. Although it was not possible to establish whether these self-diagnosed spontaneous abortions were due to flooding or other causes (e.g. a virus, an accident, intimate partner violence), they are a cause for concern. Spontaneous abortion can lead to a subsequent miscarriage if an underlying infection is not treated and managed ( Griebel *et al*., 2005). It can also contribute to chronic illness and reduced quality of life. High rates of chronic illness increase care costs ( IPPF, 2015) and hamper development ( GoB, 2003). As such there is a need to investigate the conditions under which spontaneous abortions take place during a flood, as well as to educate pregnant women on how to treat and manage spontaneous abortion through self and medical care.

It was observed that social stigma and a culture of silence around reproductive health is widespread in our research location. This is consistent with another study conducted by WHO (2012) in Bangladesh. It was observed that the participants were only willing to talk about menstrual regulation and post-abortion care privately and that some of them considered it a sin. The field research team found that talking about these topics was difficult because they were emotive. Being *chup* (silent) or not responding to the questions was common. Field research assistants honoured these silences without exerting pressure for answers in order to be consistent with the ethical policy of respecting participants’ choices/voices. As such, there was a very high non-response rate. Only 66 women out of 370 (18%) openly mentioned their health-seeking patterns for menstrual regulation and post-abortion care during the flood of 2016. To reduce such high non-response rates, research methods such as participant observation and in-depth interviews *in situ* are likely to increase women’s participation and in doing so this might provide a better insight into the challenges rural women experience during a flood. Ethnographic research could offer further insights to interpret women’s silences and non-responses. Interpretation of silences can lead to understanding of the challenges of not only married women but also of un-married girls and women who often become victims of honour killing and suicides following pregnancies that occur out of wedlock ( Fauveau & Blanchet, 1989; World Bank, 2005).

**Figure 4.**
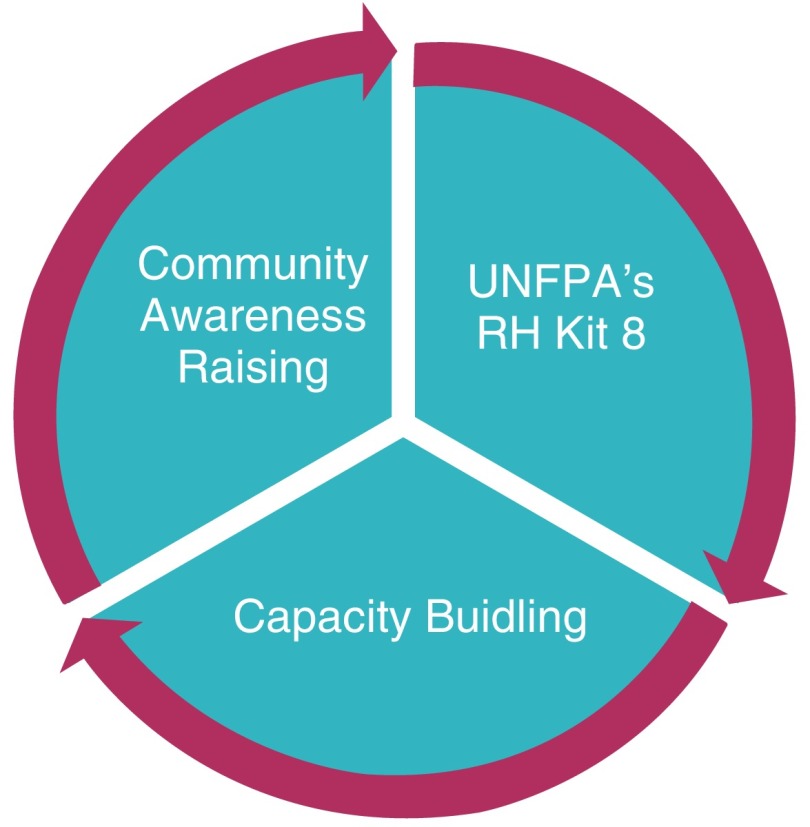
The RHCC intervention.

To conclude, intervention packages or measures which can overcome the local challenges both at community and facility levels, in an integrated fashion, are urgently needed in Bangladesh. A trial intervention package called RHCC (positioning UNFPA’s Reproductive Health Kit 8, Capacity building of untrained health workers; and Community awareness) (see Figure 4) led by the University of Leicester in collaboration with IPPF-SAR, the Upazila Health Complex management team, icddr,b and Data Management Aid, demonstrated impact by enhancing management skills and ensuring reproductive health care services were available during the flood of 2017 in Belkuchi (see Ray-Bennett *et al*., 2019 [forthcoming]). Context specific integrated interventions, such as RHCC, are essential to promote disaster resilience in the primary health care sector and to support sustainable development.

## Data availability

### Underlying data

Open Science Framework. Understanding Reproductive Health Challenges during a Flood: Insights from Belkuchi Upazila, Bangladesh. https://doi.org/10.17605/OSF.IO/VT5GW ( Ray-Bennet *et al*., 2019).

This project contains the following underlying data:

- Data from facility assessments.csv
- Raw Data for Relevant Interview Questions Asked.csv

### Extended data

Open Science Framework: Understanding Reproductive Health Challenges during a Flood: Insights from Belkuchi Upazila, Bangladesh. https://doi.org/10.17605/OSF.IO/VT5GW ( Ray-Bennet *et al*., 2019).

This project contains the following extended data:

- Data from interviews.csv (analysed data taken from participant interviews)
- Resources for menstrual regulation and post abortion care services that were assessed using the “facility structured assessment tool”.pdf
- Sample of survey questions.pdf (questions asked to each of the participants)

### Reporting guidelines

Open Science Framework: SRQR checklist for ‘Understanding Reproductive Health Challenges during a Flood: Insights from Belkuchi Upazila, Bangladesh’. https://doi.org/10.17605/OSF.IO/VT5GW ( Ray-Bennet *et al*., 2019).

Data are available under the terms of the Creative Commons Attribution 4.0 International license (CC-BY 4.0).
