# Supplementary material for: Understanding reproductive health challenges during a flood: insights from Belkuchi Upazila, Bangladesh
Source: Gates Open Res. 2019 Jun 28;3:788. Originally published 2019 Mar 11. [Version 2] doi: 10.12688/gatesopenres.12920.2 (PMC6603260; doi:10.12688/gatesopenres.12920.2)
Supplement: Peer review history

# Peer review: Reviewer response for version 2

**DOI:** 10.21956/gatesopenres.14163.r27474

**Published:** 2019-07-01

**Authors:** Nahid Rezwana

**Affiliations:** 1 Department of Geography & Environment, University of Dhaka, Dhaka, Bangladesh; 2 Bangladesh Geographical Society (BGS), Dhaka, Bangladesh

No further comments.

I confirm that I have read this submission and believe that I have an appropriate level of expertise to confirm that it is of an acceptable scientific standard.

# Peer review: Reviewer response for version 1

**DOI:** 10.21956/gatesopenres.14019.r27021

**Published:** 2019-04-23

**Authors:** Eva Luksaite

**Affiliations:** 1 School of Medicine, Keele University, Keele, UK

This is a well-written article suitable for the purpose and the format of the journal. The language of the article is accessible to different audiences which makes its ambition to impact on policy much easier to achieve. The article explores a significant and under-researched topic of the provision of reproductive health services in the period following the natural disaster in a remote and under-resourced area in Bangladesh. Various failures in the healthcare infrastructure have been identified as the main barriers for the provision of adequate menstrual regulation and post-abortion care services to vulnerable women. The findings of the article can be framed within the debates surrounding the relationship between structural violence, gender and health in a particular context in the Global South. There are several areas where the article could be enriched:

1. There are several issues which arise from the use of the chosen methodology and the authors suggest that ethnographic research methods could be used to provide more in-depth data. I could not agree more. Firstly, the article would have greatly benefited from the inclusion of women’s voices and experiences of both using healthcare services and refusing to use them. It is mentioned that some open-ended questions were included in the structured-interviews. It would be good to know if any of these questions asked women to share their experiences; if not, such questions could be included in the follow-up research projects. If such data were collected, it would be good to include direct quotations from research informants. Secondly, a long-term engagement with fewer informants which builds trust and rapport may have enabled to overcome ’the culture of silence’ which surrounds talking about reproductive health. Issues which are surrounded by stigma and taboo require more in-depth research methods, such as ethnography or the collection of life-histories and narratives. These research methods would require a much smaller ‘sample’ but would provide much richer data, especially when investigating topics such as menstrual regulation, spontaneous abortions and post-abortion care.
2. The methods section could include some more information on the interview process. It would be good to include how many field research assistants interviewed 370 women (6 field research assistants are mentioned in the acknowledgements only), how long each interview lasted and how long it took to interview 370 women. This would provide more information on the research process and the reliability of data collected.
3. More context could have been provided for readers who are not familiar with this particular area of South Asia. For instance, the article mentions different health workers (medical officer, sub-assistant community medical officers, family welfare visitors), but their different roles in the local health infrastructure remain unclear to those unfamiliar with the healthcare provision in Bangladesh. More information on the social, economic and political context of the area where research was carried out would have provided a more nuanced understanding of research findings and their implications for policy. The article states that ‘untrained health workers continue to provide menstrual regulation and post-abortion care services and this malpractice has huge consequence in decreasing patient safety, satisfaction and good practices’. This statement needs more context on who these untrained health workers are, what they do and why they are so prevalent. The presence of such untrained personnel often points to the failure of the public health system, the distrust in government services, the presence of traditional health care practices which have not been incorporated/integrated into public health infrastructure or a configuration of other social, cultural, economic and political conditions. Exploring these local conditions would provide a more in-depth understanding of pathways of care for women seeking reproductive health services. 
4. Some statements made in the article are presented as facts or realities but could have been problematised. Practices of untrained health workers are labelled as ‘malpractice’ but little explanation is provided as to why. Local health practices which fall outside of biomedicine should be explored on a case-by-case basis before being labelled as inappropriate precisely because they fall outside of the contours of biomedicine. The article reports that ‘hygiene is sometimes not maintained’ in some of the healthcare centres. What does this statement really mean? Ideas about hygiene and regulation of hygiene standards are socio-political practices which are constructed in institutional settings and implemented in locally specific ways. Peoples’ and institutions’ ideas about hygiene are different. It would be good to specify which particular set of guidelines are used here and to acknowledge that these guidelines are arbitrary and socially constructed.
5. Finally, and probably most importantly, the discussion on the status of women in the region would have been particularly welcome in order to inform the understandings of health-seeking behaviour and delays in seeking care. This includes the discussion on the economic situation of households and whether women have control over financial resources, restrictions on women’s physical mobility and whether women are allowed to travel to healthcare settings on their own, gender relations within the household and in the community and how reproductive decisions are made. In other words, the discussion on how women are embedded in local relations of power, status and kinship would contextualise research findings in significant ways.

I confirm that I have read this submission and believe that I have an appropriate level of expertise to confirm that it is of an acceptable scientific standard.

# Response

**Published:** 2019-06-21

**Authors:** Nibedita Shankar Ray-Bennett

**Response to Reviewer 2: Eva Luksaite - **School of Medicine, Keele University, Keele, UK

“This is a well-written article suitable for the purpose and the format of the journal. The language of the article is accessible to different audiences which makes its ambition to impact on policy much easier to achieve. The article explores a significant and under-researched topic of the provision of reproductive health services in the period following the natural disaster in a remote and under-resourced area in Bangladesh. Various failures in the healthcare infrastructure have been identified as the main barriers for the provision of adequate menstrual regulation and post-abortion care services to vulnerable women. The findings of the article can be framed within the debates surrounding the relationship between structural violence, gender and health in a particular context in the Global South. There are several areas where the article could be enriched:”

*- We would like to thank Eva Luksaite for reviewing our article.*

“1. There are several issues which arise from the use of the chosen methodology and the authors suggest that ethnographic research methods could be used to provide more in-depth data. I could not agree more. Firstly, the article would have greatly benefited from the inclusion of women’s voices and experiences of both using healthcare services and refusing to use them. It is mentioned that some open-ended questions were included in the structured-interviews. It would be good to know if any of these questions asked women to share their experiences; if not, such questions could be included in the follow-up research projects. If such data were collected, it would be good to include direct quotations from research informants. Secondly, a long-term engagement with fewer informants which builds trust and rapport may have enabled to overcome ’the culture of silence’ which surrounds talking about reproductive health. Issues which are surrounded by stigma and taboo require more in-depth research methods, such as ethnography or the collection of life-histories and narratives. These research methods would require a much smaller ‘sample’ but would provide much richer data, especially when investigating topics such as menstrual regulation, spontaneous abortions and post-abortion care.”

*- In regard to the reviewer’s comment that “the article would have greatly benefited from the inclusion of women’s voices and experiences of both using healthcare services and refusing to use them” – this is one of the limitations of this paper, which we have acknowledged.*

*The structured interviews did include a few open-ended questions, such as ‘Did you experience any complications after receiving menstrual regulation?’ and ‘Why did you not receive any healthcare service for these complications?’. However, the response rate was very low.*

*- We did encourage respondents to elaborate but as mentioned in the article, we also respected their culture of silence around reproductive health. In the future and for follow-up projects, ethnographic research methods are recommended to provide more in-depth data because a key part of ethnographic research would involve a rapport and trust building, which is essential. We agree that in-depth research methods would have been very useful but unfortunately, we are unable to go back in time. Furthermore, the objectives of this entire research project were different.  Nevertheless, we believe that the findings have provided valuable insights into the reproductive health challenges faced at the facility level and community level during a flood in Belkuchi Upazila. We anticipate that the research findings will act as baseline research for future research projects.*

“2. The methods section could include some more information on the interview process. It would be good to include how many field research assistants interviewed 370 women (6 field research assistants are mentioned in the acknowledgements only), how long each interview lasted and how long it took to interview 370 women. This would provide more information on the research process and the reliability of data collected.”

*- We have updated the manuscript with the details of the field research assistants who interviewed the 370 women. Please see the third paragraph in the sub-section: ‘Methods: Interviews’. As requested, we have specified how long each interview lasted (approximately 30 minutes to 2 hours) and how long it took to interview the 370 women (January-February 2019).*

“3. More context could have been provided for readers who are not familiar with this particular area of South Asia. For instance, the article mentions different health workers (medical officer, sub-assistant community medical officers, family welfare visitors), but their different roles in the local health infrastructure remain unclear to those unfamiliar with the healthcare provision in Bangladesh. More information on the social, economic and political context of the area where research was carried out would have provided a more nuanced understanding of research findings and their implications for policy.”

*- The Methodology section has been amended to provide some demography and disaster data of Belkuchi. We have also provided the role description of the health workers in a footnote.*

“The article states that ‘untrained health workers continue to provide menstrual regulation and post-abortion care services and this malpractice has huge consequence in decreasing patient safety, satisfaction and good practices’. This statement needs more context on who these untrained health workers are, what they do and why they are so prevalent. The presence of such untrained personnel often points to the failure of the public health system, the distrust in government services, the presence of traditional health care practices which have not been incorporated/integrated into public health infrastructure or a configuration of other social, cultural, economic and political conditions. Exploring these local conditions would provide a more in-depth understanding of pathways of care for women seeking reproductive health services.”

*- The untrained health workers included some of the nurses and family welfare visitors who were providing menstrual regulation without being trained. Through this project and as a result of our findings, these health workers received training* *(Ray-Bennett, Corsel and Goswami, 2019a, 2019b).*

“4. Some statements made in the article are presented as facts or realities but could have been problematised. Practices of untrained health workers are labelled as ‘malpractice’ but little explanation is provided as to why. Local health practices which fall outside of biomedicine should be explored on a case-by-case basis before being labelled as inappropriate precisely because they fall outside of the contours of biomedicine. The article reports that ‘hygiene is sometimes not maintained’ in some of the healthcare centres. What does this statement really mean? Ideas about hygiene and regulation of hygiene standards are socio-political practices which are constructed in institutional settings and implemented in locally specific ways. Peoples’ and institutions’ ideas about hygiene are different. It would be good to specify which particular set of guidelines are used here and to acknowledge that these guidelines are arbitrary and socially constructed.”

*- We agree that peoples’ and institutions’ ideas about hygiene are different. Therefore, in the section about ‘hygiene’, we have provided some further information based on the first two authors’ observations during a field visit.*

“5. Finally, and probably most importantly, the discussion on the status of women in the region would have been particularly welcome in order to inform the understandings of health-seeking behaviour and delays in seeking care. This includes the discussion on the economic situation of households and whether women have control over financial resources, restrictions on women’s physical mobility and whether women are allowed to travel to healthcare settings on their own, gender relations within the household and in the community and how reproductive decisions are made. In other words, the discussion on how women are embedded in local relations of power, status and kinship would contextualise research findings in significant ways.”

*- We appreciate these insightful comments. We feel these issues could be studied through in-depth qualitative research. This has not been the objective for this IPPF-Funded research project. The research project was geared towards how to improve the quality and availability of post-abortion care during a flood at facility level.*

# Peer review: Reviewer response for version 1

**DOI:** 10.21956/gatesopenres.14019.r26992

**Published:** 2019-04-12

**Authors:** Nahid Rezwana

**Affiliations:** 1 Department of Geography & Environment, University of Dhaka, Dhaka, Bangladesh; 2 Bangladesh Geographical Society (BGS), Dhaka, Bangladesh

This well-written paper makes a valuable contribution to the fields of disaster management, healthcare and well-being. It is helpful to understand the context of reproductive health problems and the factors affecting accessibility to healthcare centres during floods in a remote location of Bangladesh. The paper excellently presents its exciting results in paragraphs with relevant titles. However, I would like to draw the attention of the authors to the following few points which would enrich the content and explain the situations explicitly:

  

In the 'Methods' section: In the first line of the paragraph, administrative sub-divisions of the Sirajganj district is mentioned as 'sub-districts'. However, it is better to use the name of the administrative units, which is 'upazila' to avoid confusion among the readers. Every district in Bangladesh is divided into upazilas and upazila is divided into unions and then villages.
In the section 'Result: Lack of services': I would encourage the authors to explain the reasons elaborately mentioned by the family welfare visitors. The reasons are not clear. It seems that the family welfare visitors discourage the patients, and may/do not perform menstrual regulations due to their beliefs. However, as being the employee, they should be accountable to the higher authorities of the Government healthcare centres and they cannot take their decision depending on their own beliefs. Then the question arises; is the whole system of these Union Health and Family Welfare Centres involved in this mismanagement and have an influence on decreasing the service quality of the healthcare centres? Explaining this point would be very helpful.
a) In the section 'Challenges at the community level': Availability of transport should be more elaborate with statistics. Transportation is an important factor during disasters, however becomes one of the major influencing factors for women to access healthcare in rural Bangladesh (Rezwana, 2017 ^1^).
  b) Another factor; economic conditions (family and individual level) and their relationship with healthcare access could be mentioned to understand the situations in floods. Economic conditions influence the choice of healthcare centres, transport used to visit and especially time; when they would visit the centres. It is important to remember that women's healthcare access depends on their own earning status and decision-making power (Rezwana, 2017 ^1^).
  c) Education and awareness are inter-related and might have a relation with the non-response rate which was 18% in this study. It is advisable that the authors notice this point to understand the reasoning.

  

 Future plan:

 This is an excellent research, which is contemporary and engaging, however influences of 'gender' on healthcare access especially menstrual regulation during floods, is an open question for future investigation. It is important to analyse the relationship between gender identity and power relation within the family and society to understand the reasons of women's absence from the healthcare services for menstrual regulation. I look forward to the author's engagement with these issues in the future research.

I confirm that I have read this submission and believe that I have an appropriate level of expertise to confirm that it is of an acceptable scientific standard.

## References

[1] : Disasters, Gender and Access to Healthcare.2017; 10.4324/9780203701546 10.4324/9780203701546

# Response

**Published:** 2019-06-21

**Authors:** Nibedita Shankar Ray-Bennett

**Response to Reviewer 1: Nahid Rezwana - **Department of Geography & Environment, University of Dhaka, Dhaka, Bangladesh

“This well-written paper makes a valuable contribution to the fields of disaster management, healthcare and well-being. It is helpful to understand the context of reproductive health problems and the factors affecting accessibility to healthcare centres during floods in a remote location of Bangladesh. The paper excellently presents its exciting results in paragraphs with relevant titles.”

*- We would like to thank Nahid Rezwana for reviewing our article.*

“However, I would like to draw the attention of the authors to the following few points which would enrich the content and explain the situations explicitly:

1. In the 'Methods' section: In the first line of the paragraph, administrative sub-divisions of the Sirajganj district is mentioned as sub-districts.  However, it is better to use the name of the administrative units, which is 'upazila' to avoid confusion among the readers. Every district in Bangladesh is divided into upazilas and upazila is divided into unions and then villages.”

*- We have updated ‘sub-district’ to ‘upazila’ throughout the article.*

“2. In the section 'Result: Lack of services': I would encourage the authors to explain the reasons elaborately mentioned by the family welfare visitors. The reasons are not clear. It seems that the family welfare visitors discourage the patients, and may/do not perform menstrual regulations due to their beliefs. However, as being the employee, they should be accountable to the higher authorities of the Government healthcare centres and they cannot take their decision depending on their own beliefs. Then the question arises; is the whole system of these Union Health and Family Welfare Centres involved in this mismanagement and have an influence on decreasing the service quality of the healthcare centres? Explaining this point would be very helpful.”

*- As per the comment: *“In the section 'Result: Lack of services': I would encourage the authors to explain the reasons elaborately mentioned by the family welfare visitors. The reasons are not clear.”.

*The authors would like to highlight that three reasons have been provided, which were: i) absence of trained family welfare visitors; ii) religious beliefs deterred the performance of menstrual regulation procedures; and iii) motivation to increase the uptake of contraception acted as a barrier to menstrual regulation.*

*- We are unable to answer the reviewer’s question “is the whole system of these Union Health and Family Welfare Centres involved in this mismanagement and have an influence on decreasing the service quality of the healthcare centres?”. This would require further exploration. Nevertheless, the authors have amended the article to increase the clarity on the reasons described in the section ‘Result: Lack of services’.*

“3.

a) In the section 'Challenges at the community level': Availability of transport should be more elaborate with statistics. Transportation is an important factor during disasters, however becomes one of the major influencing factors for women to access healthcare in rural Bangladesh (Rezwana, 2017).”

*- We agree that transportation is an important medium to access healthcare during disasters. Unfortunately, we are unable to provide detailed information on this because we did not gather information with specific focus on transportation. Nevertheless, the authors do recognise that this could be further explored in future studies.*

“b) Another factor; economic conditions (family and individual level) and their relationship with healthcare access could be mentioned to understand the situations in floods. Economic conditions influence the choice of healthcare centres, transport used to visit and especially time; when they would visit the centres. It is important to remember that women's healthcare access depends on their own earning status and decision-making power (Rezwana, 2017)”

*- This is an excellent point. In the section on ‘Women’s care-seeking patterns’, it is mentioned that one of the main reasons for women not receiving healthcare for the menstrual regulation related complications was: “The cost of the services was too high.”. This does show that economic conditions do influence women’s choice of receiving healthcare.*

“c) Education and awareness are inter-related and might have a relation with the non-response rate which was 18% in this study. It is advisable that the authors notice this point to understand the reasoning.”

*- This is a fair comment. Since, we are unable to conclusively state that this was the reason for the high non-response rate, we have decided not to make this assumption within the article.*

“Future plan: This is an excellent research, which is contemporary and engaging, however influences of 'gender' on healthcare access especially menstrual regulation during floods, is an open question for future investigation. It is important to analyse the relationship between gender identity and power relation within the family and society to understand the reasons of women's absence from the healthcare services for menstrual regulation. I look forward to the author's engagement with these issues in the future research.”

*- We are glad that the reviewer found our research contemporary and engaging. We agree with the reviewer’s insightful comments. Analysing the relationship between gender identity and power relation within the family and society to understand the reasons of women's absence from the healthcare services for menstrual regulation, is indeed highly desirable through a qualitative approach. We would like to thank the reviewer for her time.*
